# 1696. Urinary tract infection due to *Aerococcus urinae* in children

**DOI:** 10.1093/ofid/ofad500.1529

**Published:** 2023-11-27

**Authors:** Eri Sato, Hiroyuki Iijima, Kensuke Shoji, chikara Ogimi, Akira Ishiguro

**Affiliations:** National Center for Child Health and Development, Setagaya-ku, Tokyo, Japan; National Center for Child Health and Development, Setagaya-ku, Tokyo, Japan; National Center for Child Health and Development, Setagaya-ku, Tokyo, Japan; National Center for Child Health and Development, Setagaya-ku, Tokyo, Japan; National Center for Child Health and Development, Setagaya-ku, Tokyo, Japan

## Abstract

**Background:**

*Aerococcus urinae* is a rare Gram-positive and catalase-negative coccus. *A. urinae* is primarily associated with urinary tract infection (UTI). It is responsible for urosepsis and occasionally complicated diseases, including endocarditis and osteomyelitis. The clinical presentations of adult patients with *A. urinae* have been described. However, there are very few reports in children. The clinical course of UTI due to *A. urinae* and the associated risk factors remain unclear. The aim of this study was to describe the clinical characteristics of UTI due to *A. urinae* in children.

**Methods:**

We conducted a retrospective observational study at a tertiary children’s hospital in Tokyo, Japan. We included pediatric patients (< 18 years old) who had positive urine cultures for *A. urinae* (≥ 10^4^ CFU/HPF) between June 1, 2006, and May 31, 2022. Information regarding clinical characteristics, including age, sex, underlying disease, laboratory test results, antibiotic susceptibility of *A. urinae*, treatment, and outcomes were collected from the electronic medical records.

**Results:**

A total of 22,769 urine cultures were submitted during the study period, of which *A. urinae* was isolated in 35 (0.15%). We found 17 patients who were positive for *A. urinae* with greater than 10^4^ CFU/HPF. Among them, 13 patients (76.5%) were male and 16 (94.1%) had underlying bladder dysfunction. The main clinical symptoms were fever (n=6, 35.3%), urine odor (n=4, 23.5%), and nausea (n=2, 11.8%). Five patients had diagnosed pyelonephritis due to *A. urinae*. All five patients were male, and the median age at presentation was 4.6 years (range 2.1-11.0 years). Four of them had underlying bladder dysfunction. All bacterial isolates were susceptible to the majority of antibiotics, including penicillin G, sulfamethoxazole, or ciprofloxacin. All patients had favorable outcomes following 10-14 days of ampicillin/amoxicillin-based antimicrobial therapy.

**Conclusion:**

*A. urinae* was isolated in urine cultures primarily in male patients with underlying bladder dysfunction. All of the outcomes of antibiotic treatment were favorable.

**Disclosures:**

**Kensuke Shoji, MD, PhD**, AstraZeneca K.K.: Honoraria|Gilead Sciences, Inc.: Honoraria|KYORIN Pharmaceutical Co.,Ltd.: Honoraria|Meiji Seika Pharma Co., Ltd.: Honoraria|Nippon Becton Dickinson Company, Ltd.: Honoraria|Novartis Pharma Co., Ltd.: Honoraria|Viatrs, Inc: Honoraria **chikara Ogimi, MD**, bioMerieux Japan Ltd.: Honoraria|Horiba: Honoraria|Pfizer: Honoraria

